# Reproductive endocrinology of endangered black-footed ferrets: implications for conservation breeding

**DOI:** 10.1093/conphys/coaf002

**Published:** 2025-02-13

**Authors:** Daphne A Arguelles, Phoebe D Edwards, Ayesha Beyersbergen, Melissa M Holmes, Gabriela F Mastromonaco

**Affiliations:** Department of Psychology, University of Toronto Mississauga, 3359 Mississauga Rd, Mississauga, ON L5L 1C6, Canada; Department of Psychology, University of Toronto Mississauga, 3359 Mississauga Rd, Mississauga, ON L5L 1C6, Canada; Reproductive Sciences, Toronto Zoo, 2000 Meadowvale Rd, Toronto, ON M1B 5K7, Canada; Reproductive Sciences, Toronto Zoo, 2000 Meadowvale Rd, Toronto, ON M1B 5K7, Canada; Department of Psychology, University of Toronto Mississauga, 3359 Mississauga Rd, Mississauga, ON L5L 1C6, Canada; Reproductive Sciences, Toronto Zoo, 2000 Meadowvale Rd, Toronto, ON M1B 5K7, Canada

**Keywords:** Carnivore, conservation breeding, cortisol, oestradiol, faecal hormone metabolites, progesterone, reproduction, stress, testosterone

## Abstract

The black-footed ferret (*Mustela nigripes*) is an endangered North American mustelid. This species is bred in managed care with the goal of reestablishing wild populations. However, individual ferrets in the conservation breeding programme have variable reproductive success. We monitored faecal steroid hormone metabolite profiles of 22 black-footed ferrets across two breeding seasons to examine whether endocrine factors were associated with successful reproduction. Among successfully whelping females, faecal progesterone metabolite concentrations were higher (*P* = 0.04) and faecal cortisol metabolite (FCM) concentrations were marginally higher (*P* = 0.07) in the late luteal phase compared to females who did not whelp (likely pseudopregnant). Effect sizes suggested that, in successfully whelping females, faecal oestradiol metabolite levels were higher in the follicular phase and FCM levels lower in the early luteal phase, but with high variation and lack of statistical significance. We speculate that this variation may be because male causes of reproductive failure account for some of these cases of pseudopregnancy. Among males, individuals that failed to successfully copulate had lower faecal testosterone metabolite concentrations than successful sires (*P* = 0.01). However, males who copulated but failed to sire a litter did not differ from successful sires in testosterone metabolite concentrations. Comparisons of sperm morphology between successful and unsuccessful sires were statistically underpowered, hence poor sperm quality could not be ruled out as a possible cause of these post-copulatory reproductive failures. Our data suggest that individuals who fail to reproduce in managed care are not experiencing chronic stress, based on FCM levels, although changes in females during the early luteal phase warrant further investigation. While male post-copulatory reproductive failure was not associated with deficiencies in sex hormone production, males that fail to copulate could potentially be targeted for testosterone supplementation.

## Introduction

Black-footed ferrets (*Mustela nigripes*) are an endangered mustelid native to the North American grasslands. This species was thought to be extinct until a small population was discovered in 1981. Due to disease (sylvatic plague, canine distemper), the population dwindled to only 18 individuals. As an emergency measure for the recovery of this species, the 18 individuals were brought into captivity in 1987 with seven of them becoming founders of a conservation breeding programme, which has continued until present (Williams *et al.*, 1991; Biggins *et al.*, 1998; Santymire and Graves, 2019). Currently, there is a breeding population of ~300 black-footed ferrets across six facilities managed by the US Fish and Wildlife Service and the Association of Zoos and Aquariums (Santymire and Graves, 2019). Each year, a subset of the offspring born in this programme are released back into the wild. Yet, black-footed ferret populations still face considerable challenges from habitat destruction, depletion of their primary prey (prairie dogs, *Cynomy*s spp.), disease, predation and reduced levels of reproduction, potentially as a result of inbreeding depression (Forrest *et al.*, 1988; Jachowski *et al.*, 2011; Santymire *et al.*, 2014). Hence, efforts are ongoing to breed and reintroduce individuals into the wild until natural populations can become self-sustaining (Santymire and Graves, 2019).

To maintain the genetic diversity of managed populations, individuals in the breeding programme are cycled through different pairings based on mean kinship values to minimize inbreeding and encourage breeding between under-represented individuals. Adult ferrets are individually housed and introduced to potential mates while females are in oestrus. However, individual black-footed ferrets in the conservation breeding programme have variable reproductive success. In a study across black-footed ferret breeding facilities over three breeding seasons, 55–58% of males of prime breeding age failed to sire a litter each season (Wolf *et al.*, 2000a). Within that study, causes of reproductive failure were identified including male behaviour (34% of unsuccessful sire cases; improper breeding positioning and behaviour towards estrous females) and male physiology (41% of unsuccessful sire cases; including underdeveloped testes and copulation with no sperm observed in the post-coital lavage).

Post-copulatory issues have been documented as well. In 18% of cases in the above study, sperm was present but there was no resulting pregnancy (Wolf *et al.*, 2000a). Failure of the pregnancy to progress may be related to structurally abnormal sperm; black-footed ferret males of prime breeding age have ~50–60% structurally normal sperm per ejaculate (Wolf *et al.*, 2000b), which is on the borderline of teratospermia (<60% morphologically normal sperm per ejaculate; Howard and Wildt, 2009). Other estimates have been even lower (20% structurally normal sperm; Howard and Wildt, 2009) and suggestive that sperm quality is decreasing over time in this highly managed population (Santymire *et al.*, 2019). Generally, teratospermia is widely documented in endangered carnivores and is suspected to be related to low genetic variability and inbreeding (Pukazhenthi *et al.*, 2006; Howard and Wildt, 2009; Santymire *et al.*, 2019).

Female post-copulatory reproductive issues may be involved, but the underlying physiology is not well understood. Following ovulation, it can be difficult to determine whether a mated female has become pregnant because black-footed ferrets, like many other carnivores, exhibit pseudopregnancy, in which progesterone secretion from the corpus luteum is maintained beyond the second trimester or for the full gestation length. Hence, pregnant and pseudopregnant females show comparable oestradiol and progesterone profiles during the breeding period (Brown, 1997), until the late luteal phase when progesterone levels in pregnant females increase above those of pseudopregnant females (Young *et al.*, 2001). Females who exhibit oestrus but do not whelp have generally been presumed to be pseudopregnant, although it is possible that females could become pregnant and lose foetuses, undetected. No hormonal predictors of which mated females will become pregnant or pseudopregnant have been identified.

The aim of our study was to improve our understanding of the physiological factors associated with successful reproduction in black-footed ferrets by examining multiple steroid hormone profiles across the breeding season and how individuals with different reproductive outcomes may differ within reproductive state (follicular phase, oestrus, early/late luteal phase and postpartum). Some endocrine correlates of reproductive success have been previously identified in this species. Maternal faecal oestradiol concentrations in the late luteal phase have been found to be positively correlated with litter size (Young *et al.*, 2001). In males, testosterone production is seasonally modulated, increasing in the spring ~2 months before females enter oestrus and declining in the summer as the breeding season ends (Brown, 1997). Testosterone levels are comparable among 1- to 5-year-old males but decline marginally at age 6–7 (Wolf *et al.*, 2000b). A recent study using hair cortisol measures found that hair cortisol concentrations did not differ in pregnant and pseudopregnant females overall; however, hair cortisol concentrations were higher in males who successfully sired litters than males who did not at one particular study site (Santymire *et al.*, 2021).

We expanded upon this work by using non-invasive faecal sampling of individual black-footed ferrets across two breeding seasons. We documented oestradiol and progesterone metabolite concentrations in females, testosterone metabolite concentrations in males and cortisol metabolite concentrations in both sexes throughout the mating, pregnancy and post-natal period. We additionally used this data to assess progesterone/oestradiol ratio (P:E ratio) across time points, as a high P:E ratio (or low E:P ratio) has been associated with successful blastocyst implantation in other species (Gidley-Baird *et al.*, 1986; Thorpe *et al.*, 2013). Further, we compared records of sperm morphology data among successful and unsuccessful sires. In addition to the conservation value of the breeding programme, managed breeding provides the exceptional opportunity for the in-depth study of the reproductive endocrinology of these rare carnivores, with sampling resolution and knowledge of individual identity, reproductive state and reproductive outcome that would be difficult or impossible to obtain in a field context.

## Materials and Methods

### Classification of reproductive state

Black-footed ferrets are seasonal breeders, with the majority of mating activity occurring in April, and the majority of whelping in May–June (Williams *et al.*, 1991). Ovarian cycle phase is determined by vaginal cytology (>90% cornified cells indicative of oestrus), and following pairing, a post-coital vaginal lavage is performed to test for the presence of sperm (Williams *et al.*, 1991; Young *et al.*, 2001). Pro-oestrus lasts 14–21 days, and females can remain in oestrus for 30–40 days if they are not mated (Williams *et al.*, 1991; Young *et al.*, 2001). In accordance with prior work, we consider post-mating Days 3–11 the early luteal phase and Days 12–40 the late luteal phase, as implantation is expected to occur around Day 12–13 of pregnancy (Young *et al.*, 2001). Gestation is ~42 days (Williams *et al.*, 1991). Black-footed ferrets do not exhibit delayed implantation (Thom *et al.*, 2004). If a female is not impregnated in the spring or loses her litter, she may exhibit a second oestrus later in the summer, but this is generally uncommon (16% of females without litters displayed a second oestrus; Williams *et al.*, 1991).

### Animal housing and faecal sample collection

All procedures were conducted in accordance with the Toronto Zoo Animal Care and Research Committee guidelines. Black-footed ferrets located in the Toronto Zoo conservation breeding programme facility were monitored across two breeding seasons (29 March–12 June 2022 and 13 April–10 July 2023). Ferrets are kept at a facility that is not open or accessible to the public, where they were housed indoors at ambient conditions of 19–22°C, maintained on a natural photoperiod, and fed a Toronto Zoo small-carnivore diet as previously described (Mackie *et al.*, 2020). All animals in this study were born in the conservation breeding programme. All animals are single-housed except for bouts of pairing during the mating period, as this species is solitary and territorial in the wild (Richardson *et al.*, 1987). In 2022, the sampling cohort included eight females and nine males, and in 2023, it included eight females and seven males. Of these individuals, 10 were present during both years of the study, and 12 for a single year of the study (22 distinct individuals total). In 2022, two 5-year-old males were not introduced to any females due to their advanced age, but these individuals were still monitored hormonally. All other animals were 1–4 years old and given opportunities for breeding.

Over each breeding season, faecal samples were collected by keepers three times per week during routine cleaning. Keepers were asked to collect fresh samples that did not appear to be soiled with urine. On each collection day, samples were collected from a random five males and five females. If keepers could not obtain a sample from a given individual, they were asked to collect it from another individual of the same sex or collect it the next day. Sample collection ended for the males after all breeding pairings were complete, and sample collection ended for the females after all females were postpartum (or past their expected due date). Because due dates varied by individual, the postpartum sample collection range varied, with a longer collection time period for females who had earlier due dates. In total, 572 faecal samples were collected, with 345 of them from females and 227 from males. Labelled plastic bags with individual faecal samples were stored at −20**°**C until sample extraction.

### Faecal hormone analysis

Faecal samples were thawed to room temperature and weighed in glass scintillation vials. Samples were extracted in 80% methanol at a ratio of 0.50 g faeces to 5 ml methanol. The extraction mixtures were vortexed for ~5 s, placed on an orbital shaker at 100 RPM for 1 h, and then centrifuged for 10 min at 3500 RPM. The extract supernatant was then transferred to a clean labelled vial and stored at −20**°**C until analysis.

Concentrations of faecal metabolites of cortisol, oestradiol, pregnane and testosterone were analysed using enzyme immunoassays (EIAs). The antibody used in the cortisol EIA has been previously validated for black-footed ferrets by restraint and ACTH challenge (Young *et al.*, 2001). This work demonstrated that faecal samples reflect circulating cortisol levels 20–44 h prior, and that faecal cortisol and corticosterone metabolites follow a similar temporal pattern in this species (Young *et al.*, 2001). Oestradiol and progestin EIAs used previously for this species (different antibodies than in the current study) have been biologically validated by comparison with the known reproductive state of the studied individuals (Young *et al.*, 2001). To determine if the sex hormone EIAs that we used reflected biologically meaningful changes in the animals, we similarly compared the patterns of sex hormone metabolite excretion to known reproductive state and to the patterns found in this prior work.

The EIA laboratory methods used in this study have been previously described in Kummrow *et al.* (2011) and were as follows: microtitre plates (Nunc Maxisorp, VWR) were coated with antisera (C. Munro, University of California, Davis, CA) diluted in a coating buffer and incubated overnight at 4°C. Antisera used were anti-cortisol antibody (R4866), anti-oestradiol antibody (R008), anti-pregnane antibody (monoclonal CL425) and anti-testosterone antibody (R156/7). Unbound antiserum was washed from coated plates with 0.02% Tween 20 wash solution using a microplate washer (BioTek Instruments). Standards, controls and diluted faecal extracts were added to the plates in duplicates, at a volume of 50 μl per well for all. Standards used were hydrocortisone (Sigma H0135; 3.9–1000 pg/50 μl), 17 β-oestradiol (Sigma E8875; 0.98–250 pg/50 μl), progesterone (Sigma P0130; 0.78–200 pg/50 μl) and testosterone (Steraloids Inc. A6950; 2.4–625 pg/50 μl). This was followed by 50 μl of horseradish peroxidase conjugate diluted in assay buffer at the following concentrations: cortisol-HRP, 1:33 500; oestradiol-HRP, 1:30 000; progesterone-HRP 1:40 000; and testosterone-HRP 1:20 000. Controls were laboratory stocks of pooled faecal extracts obtained from a variety of species (Kummrow *et al.*, 2011) run at established dilutions resulting in ~30% binding (low control) and ~70% binding (high control). Plates were incubated for 2 h at room temperature, then washed again, whereafter 100 ml of substrate solution (50 mM citrate, 1.6 mM hydrogen peroxide and 0.4 mM 2,2′-azino-di-(3-ethylbenzthiazoline sulfonic acid) diammonium salt, pH 4.0) was added to each well. Absorbance was measured using a spectrophotometer at a setting of 405 nm (BioTek Instruments).

Serial dilutions of pooled extracts were run for each assay type to determine the appropriate dilution of samples in phosphate buffer and produce parallel curves to that of the appropriate standard. Resulting dilutions of methanol extracts were as follows: 1:8 for oestradiol assays, 1:50 for pregnane assays, 1:40 for testosterone assays and 1:5 for cortisol assays. For the pregnane and testosterone assays, an aliquot of extract was directly diluted with phosphate buffer. For the oestradiol and cortisol assays, because the dilutions required were highly concentrated, an aliquot of extract was dried down overnight and reconstituted in phosphate buffer, such that the amount of methanol added to EIAs was <10% per sample. The inter-assay coefficients of variation (CVs) were calculated using high- and low-concentration controls run on each plate. High and low CVs were 15.25 and 15.79% for oestradiol plates, 8.24 and 15.43% for pregnane plates, 10.92 and 15.26 for testosterone plates and 7.12 and 15.39% for cortisol plates, respectively. Results were reported as nanogramme hormone per gramme faeces (ng/g).

### Sperm collection and morphology analysis

We compared sperm morphology from males that successfully sired litters and males that successfully mated (positive post-copulatory sperm checks) but failed to sire a litter. Annual records of sperm morphology data were obtained from the Toronto Zoo’s managed population of black-footed ferrets, and we analysed data from the first year that both detailed breeding summary reports and sperm morphology reports occur, until present (2015–23). Each year, semen samples were collected once from each male in the breeding programme during the spring (typically 24 March–28 April, with two samples collected on 23 February and 24 May), and microscopically assessed to document any morphological defects. Semen was collected using electroejaculation from 2015 to 2017 and urethral catheterization from 2018 to 2023 (for details on collection methodology see Mackie *et al.*, 2020). Sample collection was not always successful, with an average of 4.4 samples a year collected via catheterization and an average of 2.7 samples a year collected via electroejaculation. Overall, as reported in Mackie *et al.* (2020), catheterization resulted in a greater number of successfully collected samples, and sperm morphology following catheterization did not appear to differ compared to prior reports on samples collected by electroejaculation (Santymire *et al.*, 2019; Mackie *et al.*, 2020). In total, there were 21 semen samples that were used for sperm morphology analysis from males with positive sperm checks from 2015 to 2023. For each sample, a small volume of semen was smeared on a microscopic slide, fixed in 1% paraformaldehyde and stained (Spermac Stain, FertiPro NV, Belgium). From the slides, percentage of structurally normal sperm per sample was calculated, as well as percentages of specific major morphological defects. The major sperm morphological defects assessed were: head abnormalities (including macrocephalic, microcephalic, bicephalic, pyriform, vacuole or knobbed), mid-piece abnormalities, proximal droplets and tail abnormalities (including coiled tails or biflagellate sperm). Acrosomal defects were recorded for some samples (*n* = 13), but not all, and so sample size was not sufficient to assess acrosomal defects separately.

### Statistical analysis

Reproductive outcomes for females were assigned as: whelp, did not whelp (DNW) and no oestrus. Reproductive states for females were assigned as: follicular (pro-oestrus, at least 2 days before oestrus as determined by vaginal cytology), oestrus (1 day prior to >90% cornified cells, to 5 days after, during which time ferrets are mated), early luteal (up to post-mating Day 11), late luteal (post-mating Day 12-due date) and postpartum (after due date or anticipated due date for DNW females). A single non-oestrus female was dropped from the statistical analysis due to being the only individual with this reproductive outcome. The association between hormone metabolite concentrations and reproductive correlates in females was analysed using linear mixed effect models, with each hormone as the response, animal ID nested within year as random effects and reproductive outcome, reproductive state and their interaction as fixed effects. The differences between outcomes within reproductive state were analysed with a *post hoc* Tukey test, using a simple pairwise comparison between reproductive outcomes within each reproductive state. Hormone data (and P:E ratio data) were log transformed so that model residuals would better fit assumptions of normality. The same analysis was repeated within whelping females, replacing reproductive outcome with litter size, and keeping reproductive state and the interaction as fixed effects and animal ID within year as random effects.

Reproductive outcomes for males were assigned as: not mated (two older males who were not paired with females; 5- to 7-year-old males tend to have lower testes volumes and less structurally normal sperm; Wolf *et al.*, 2000b), failed to mate (failed to copulate or negative sperm check), successfully sired and female did not whelp (successful copulation and positive sperm check, but the female did not become pregnant). The association between hormone metabolite concentrations and reproductive correlates in males were analysed using linear mixed effect models, with each hormone as the response, animal ID nested within year as random effects and reproductive outcome and study day as fixed effects. A *post hoc* Tukey test was used to compare differences among reproductive outcomes. Study day in males was relative to the first oestrus that breeding season (i.e. the date of the first female’s oestrus that year was considered Day 0).

Sperm morphology data, expressed as percentages of a given structural defect, were compared using the Wilcoxon rank sum test between successful sires and males who successfully mated (positive post-copulatory sperm check) but did not sire a litter. Because of the generally low sample sizes for sperm morphology data, power analyses were additionally performed. Data were analysed using R version 4.2.2 (R Core Team, 2022), models were built with the package ‘nlme’ (Pinhiero *et al.*, 2022) and data were visualized with ‘ggplot 2’ (Wickham, 2016) and ‘ggsankey’ (Sjoberg, 2023). Power analyses for Wilcoxon rank sum tests were conducted with the package ‘MKpower’ (Kohl, 2023).

## Results

### Reproductive outcomes

**Figure 1:**
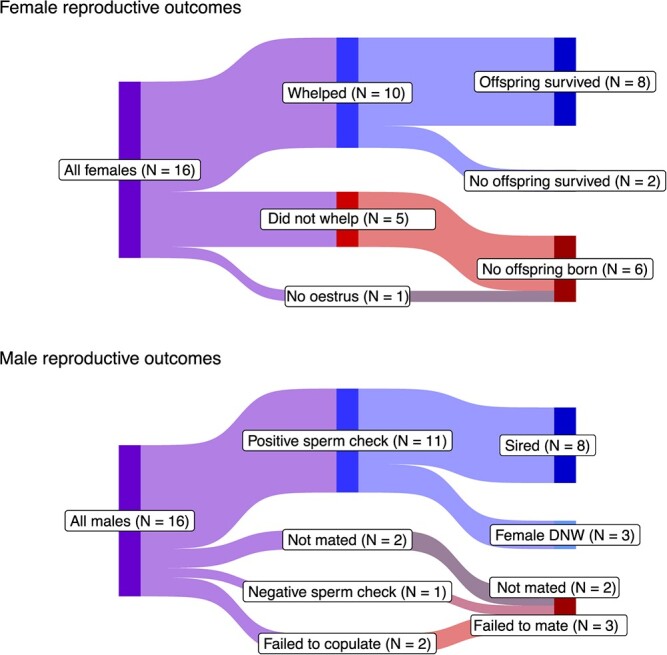
All black-footed ferret reproductive outcomes across two breeding seasons, with females displayed in the top panel and males in the bottom

The occurrences of all reproductive outcomes for females and males are shown in Fig. 1 and Supplementary Table S1. In 2022, females came into oestrus from 27 March to 10 April, and in 2023, females came into oestrus later in the season, from 2 to 16 May. In 2022, six out of eight females whelped, and in 2023, four out of eight females whelped. Of the six total females that did not reproduce, one never ovulated and the other five did not whelp, and were presumably pseudopregnant (entered oestrus and sperm was detected during post-coital vaginal lavage). Two of the females that did not whelp had been paired with the same male in different years. Of the females that successfully whelped, within each year, there was one female that had no surviving weaned offspring, and these were different females. The average litter size was 2.5, and the average number of offspring that survived to weaning age per litter was 1.7.

Among males, in 2022, aside from the two older males who were not bred, six out of seven males had positive sperm checks following post-coital vaginal lavage. Of these six, four successfully sired litters. In 2023, out of seven males, two young males (1-year-old) did not successfully copulate with receptive females. Of the remaining five males, all had positive sperm checks, and four successfully sired litters.

### Endocrine comparisons of pregnancy outcomes in females

**Figure 2:**
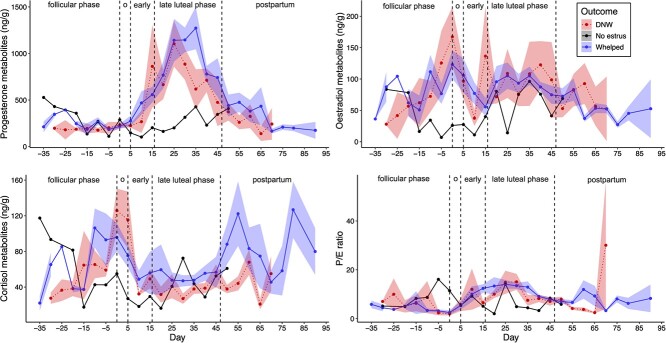
Faecal hormone metabolite concentrations in female black-footed ferrets across the breeding period. Days are relative to oestrus at Day 0, and vertical dashed lines separate periods into follicular, oestrus/mating (o), early luteal, late luteal and postpartum. For the single non-oestrus female, Day 0 was assigned as the most common day of oestrus during that year. Successfully whelping females are represented by blue solid lines, females who did not whelp (DNW) by red dotted lines, and the non-oestrus female in black. Points are mean hormone metabolite concentrations by category for a given day. For data visualization only, day of collection was rounded to the nearest fifth day. Ribbons show standard errors of the mean.

Faecal hormone metabolite concentrations and the P:E ratio across the breeding season in females are visualized in Fig. 2. All results of mixed effect models and *post hoc* tests analysing female hormone metabolite levels by reproductive outcome and state are shown in Supplementary Table S2. Relative to the follicular phase, faecal progesterone metabolite (FPM) concentrations were elevated during the early luteal (*P* = 0.004) and late luteal (*P* < 0.001) phases, faecal oestradiol metabolite (FEM) concentrations were marginally elevated during oestrus (*P* = 0.06) and the P:E ratio was elevated during the late luteal phase (*P* < 0.001; Supplementary Table S2). Faecal cortisol metabolite (FCM) concentrations were marginally elevated during oestrus (*P* = 0.09) and during the late luteal phase (*P* = 0.05).

**Figure 3:**
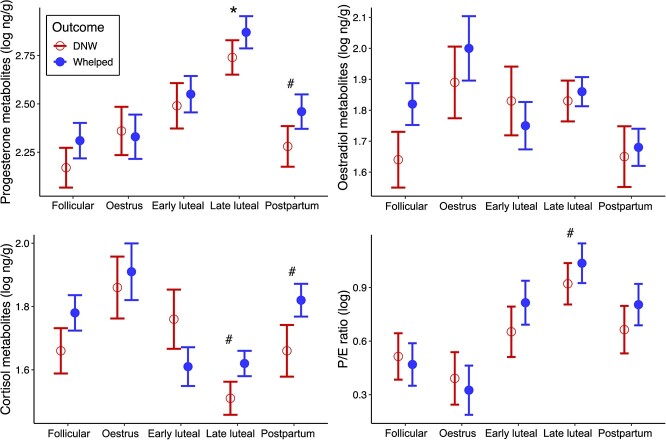
Model-estimated means (hormone log ng/g) for each reproductive outcome by reproductive state in female black-footed ferrets. Error bars indicate the standard error of the model estimate. Successfully whelping females are represented by blue closed circles and females who did not whelp (DNW) by red open circles. Statistically significant differences between groups (*P* < 0.05) are denoted by * and marginal significance (*P* < 0.10) are denoted by ^#^. *Post hoc* testing indicated that whelping and DNW females significantly differed in FPM levels during the late luteal phase (*P* = 0.04), marginally differed in FPM levels during the postpartum phase (*P* = 0.06) and marginally differed in FCM levels during the late luteal (*P* = 0.07) and postpartum phase (*P* = 0.09).

There was no main effect of reproductive outcome on any of the hormone metabolite concentrations. However, *post hoc* tests indicated that within reproductive state, differences in reproductive outcome emerged (Fig. 3). During the late luteal and postpartum periods, DNW females had reduced FPM concentrations relative to successfully whelping females (β = −0.13 ± 0.06, *P* = 0.04 and β = −0.18 ± 0.09, *P* = 0.06, respectively; Fig. 3). While overall there were no statistically significant *post hoc* differences in FEM concentrations by reproductive outcome, within the follicular phase, the effect size of the comparison of whelping and DNW females was equally as high as the comparison of FPM concentrations in the postpartum phase above, albeit with greater variation (β = −0.18 ± 0.11, *P* = 0.14). The other reproductive states showed no notable effect of reproductive outcome on FEM levels.

With respect to FCM concentrations, DNW females had marginally reduced FCM levels during the late luteal (β = −0.12 ± 0.06, *P* = 0.07) and postpartum (β = −0.17 ± 0.09, *P* = 0.09; Fig. 3) phases relative to successfully whelping females. Although these results were similar to the patterns in state-specific FPM differences by reproductive outcome, a linear model showed no direct correlation between log FPM and FCM concentrations (*P* = 0.71).

Reproductive state-specific differences in P:E ratio by reproductive outcome generally recapitulated FPM differences: successfully whelping females had a marginally greater P:E ratio in the late luteal phase (β = −0.12 ± 0.06, *P* = 0.06) when FPM concentrations were at their maximum (Fig. 2, 3). Finally, within whelping females, no main effect of litter size was detected on any of the faecal hormone metabolite concentrations (progesterone: β = 0.01 ± 0.03, *P* = 0.83; oestradiol: β = 0.02 ± 0.04, *P* = 0.63; cortisol: β = −0.03 ± 0.04, *P* = 0.41; P:E ratio: β = 0.002 ± 0.03, *P* = 0.93).

Of the females that were represented in both years of the study (*n* = 5), two of these females successfully whelped both years (Supplementary Table S1). Of the three females with different reproductive outcomes by year, individual comparisons of faecal hormone metabolites each year can be found in the Supplementary Materials (Fig. S1).

### Endocrine comparisons of reproductive outcomes in males

**Figure 4:**
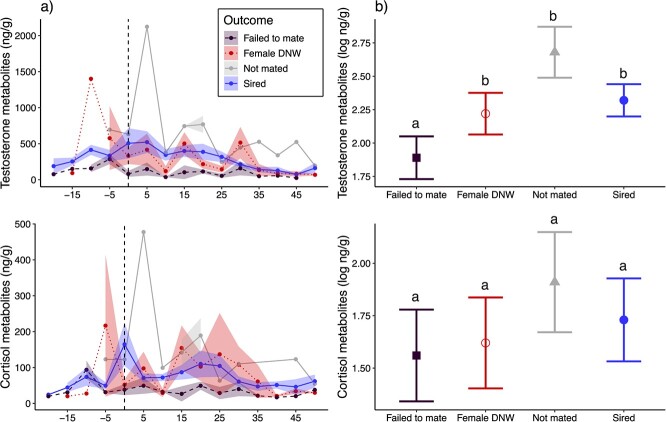
Faecal hormone metabolites in male black-footed ferrets. (a) Faecal testosterone and cortisol metabolites over the breeding period. Day 0 represents the date of the first female oestrus of the year. Males that successfully sired a litter are represented by blue solid lines, males that successfully mated but females did not whelp (DNW) as red dotted lines, males that failed to mate (including failure to copulate with a female and negative sperm check after mating) as purple dashed lines, and not mated (older males) as light grey lines. (b) Model-estimate means and standard error for each reproductive category. *Post hoc* testing indicated that males who failed to mate had lower FTM levels than successful sires (*P* = 0.01), males who were not mated (older males; *P* = 0.004) and marginally lower levels than males who mated but the female did not whelp (DNW; *P* = 0.09). The other three groups did not differ among each other in FTM levels, and FCM levels did not vary by reproductive outcome. Significance is denoted by letters, with categories that share the same letter not significantly differing from each other.

Male faecal hormone metabolite concentrations over the breeding season are displayed in Fig. 4a. There was a strong, negative association between study day and faecal testosterone metabolite (FTM) concentrations, where FTM levels decreased later in the breeding season as study days advanced (β = −0.01 ± 0.00, *P* < 0.001). When male reproductive outcomes were compared, relative to the males that failed to mate, successful sires had higher FTM concentrations (β = 0.43 ± 0.15, *P* = 0.01), as did the older males that were not mated (β = 0.79 ± 0.21, *P* = 0.004). Males who mated but the female did not whelp had only marginally higher FTM concentrations than the males who failed to mate (β = 0.33 ± 0.18, *P* = 0.09; Fig. 4b). *Post hoc* tests additionally revealed that successful sires did not differ from the unmated older males (β = 0.36 ± 0.18, *P* = 0.26) nor the males where the female did not whelp (β = −0.10 ± 0.15, *P* = 0.89; Fig. 4b). FCMs similarly displayed a negative association with study day, decreasing later in the breeding season (β = −0.01 ± 0.00, *P* < 0.001; Fig. 4a). FCM concentrations did not differ among any of the reproductive outcomes (all *P* > 0.10; Fig. 4b). A linear model revealed a significant positive correlation between log testosterone and cortisol metabolites (*P* < 0.001, R^2^ = 0.39).

### Sperm morphology comparisons

When comparing the overall percentage of structurally normal sperm, there was no difference between successful and unsuccessful sires (*P* = 0.14). Successful sires had an average of 39.8% structurally normal sperm (range: 24–56%), while unsuccessful sires had an average of 46.0% (range: 11–61%). When it came to specific structural abnormalities, there was no difference in the percentage of head abnormalities (*P* = 0.33, mean successful = 1.6%, mean unsuccessful = 3.2%), mid-piece abnormalities (*P* = 0.13, mean successful = 1.4%, mean unsuccessful = 2.5%), proximal droplets (*P* = 0.12, mean successful = 1.2%, mean unsuccessful = 2.7%) nor tail abnormalities (*P* = 0.30, mean successful = 9.7%, mean unsuccessful = 14.4%).

However, power analyses indicated that the sperm morphology data analysis was severely underpowered (*n* = 21, 9 successful sires, 12 unsuccessful). Based on group averages and standard deviations, the statistical power of detecting a significant difference with 10 samples per group for overall percentage of structurally normal sperm was only 0.15, i.e. only a 15% chance of detecting a true positive (when α = 0.05). Hence, sperm morphology data should be considered descriptive only.

## Discussion

We quantified faecal hormone metabolites across two breeding seasons in endangered black-footed ferrets in a conservation breeding programme. We found that, predictably, hormone metabolites varied by reproductive state, but they additionally varied by ultimate reproductive outcome during some time periods. In successful females, FPM concentrations were elevated (*P* < 0.001) and FCM concentrations marginally elevated (*P* = 0.07) in the late luteal phase compared to females who did not whelp. Concentrations of FTM did not differ among successful sires, older males and males who mated but did not successfully sire a litter. However, males that failed to approach and mate with a female had lower FTM concentrations than successful sires (*P* = 0.01) and the older males who were not mated (*P* = 0.004). Elevated FCMs were not associated with reproductive failure in either sex, and FCMs were positively associated with FTMs in males.

Differences in faecal hormone metabolites by reproductive state met basic assumptions of reproductive biology and are aligned with prior work in black-footed ferrets. FPM concentrations were elevated during the early luteal (*P* = 0.004) and late luteal (*P* < 0.001) phases, consistent with prior findings using a progestin EIA on black footed-ferret faecal samples (Young *et al.*, 2001) and a progestogen radioimmunoassay (RIA) on black-footed ferret faecal samples (Brown, 1997). We found that FEM concentrations were marginally elevated during oestrus (*P* = 0.06), and similarly, prior results using an oestradiol EIA (Young *et al.*, 2001) and oestradiol RIA (Brown, 1997) determined that oestradiol concentrations were positively correlated with the percentage of superficial epithelial cells in the vaginal lavage (an indicator of oestrus) in black-footed ferrets. In male black-footed ferrets, we detected a decrease in FTM later in the breeding season (*P* < 0.001), which is aligned with reports from testosterone RIAs (Brown, 1997). Similarly, a study using the same testosterone EIA as our current study detected a decrease in FTM from the breeding to post-breeding season (Santymire *et al.*, 2020). Based on this data, we conclude that the sex hormone EIAs that we used in this study detected biologically meaningful changes in sex hormone metabolites in black-footed ferret faecal samples.

Our data revealed that FPM concentrations were elevated in successfully whelping females compared to DNW females during the late luteal phase (in agreement with Young *et al.*, 2001) and marginally during the postpartum period (*P* = 0.06). The postpartum difference may be associated with lactation in the successfully whelping females. Lactation has been linked to elevated progesterone concentrations in some other mustelids such as domestic ferrets (*Mustela furo*; Proháczik *et al.*, 2011) and European mink (Nagl *et al.*, 2015), where concentrations remain high during nursing and drop as offspring begin to wean. Another mustelid, the wolverine (*Gulo gulo*), similarly displays increased progesterone concentrations during the luteal phase and postpartum, relative to basal/non-breeding levels, although there are no progesterone concentration differences between pregnant and pseudopregnant females (Dalerum *et al.*, 2006; Bateman *et al.*, 2023). Importantly, wolverines have delayed implantation while black-footed ferrets do not (Thom *et al.*, 2004,), and the lack of progesterone decline during these periods in the pseudopregnant female wolverine could be related to facilitating delayed implantation, for which the continued activity of progesterone and the corpus luteum is critical (Murphy *et al.*, 1980; Mead, 1986; Huang *et al.*, 1993). In contrast, without delayed implantation in the black-footed ferret, progesterone concentrations drop in the late luteal phase in DNW females.

FCM concentrations varied during the late luteal and postpartum periods, with successfully whelping females having marginally higher levels during both time periods than DNW females (*P* = 0.07 and *P* = 0.09, respectively). We ascribe this potential increase in FCM concentrations in whelping females to the metabolic changes of pregnancy and lactation, and the role of cortisol in foetal developmental processes. Cortisol has been found to be elevated during late pregnancy and lactation in many other mammals (reviewed by Edwards and Boonstra, 2018; Stead *et al.*, 2022,), and it is sensical that FCM concentrations would be elevated during these times in truly pregnant individuals compared to pseudopregnancy. FCM concentrations were marginally elevated during oestrus in female black-footed ferrets overall (*P* = 0.09), but with no difference between successful and DNW females (*P* = 0.71). While this increase could be related to the psychosocial stress of being paired with a new mate, elevated glucocorticoids during oestrus or ovulation have been detected in some other mammals as well (e.g. Ziegler *et al.*, 1995; Kersey *et al.*, 2011; Fanson *et al.*, 2014). While there may be a functional reason for glucocorticoid increase during oestrus, this increase is not consistent across mammals and the functional role (if any) remains to be determined. The effect size of the whelping to DNW female comparison in FCM concentrations during the early luteal period was comparable to that of the late luteal and postpartum periods, but with DNW females having elevated FCM concentrations during the early luteal period (Supplementary Table S2). However, variation was high and this difference was not statistically significant (*P* = 0.20). Similarly, the greatest effect size in FEM comparisons among reproductive outcomes was in the follicular period, with successfully whelping females having higher FEM concentrations prior to oestrus, but not significantly so (*P* = 0.14). These results suggest that there is too much variation in FCM concentrations within reproductive outcome to make meaningful distinctions. It is important to consider that the DNW category of females presumably includes pregnancies that failed to progress due to both female and male reproductive issues. Overall, the female FCM data demonstrated that failure to whelp was not associated with increased FCMs, as generally no differences were found between whelping and DNW females. The only marginal differences in FCM concentrations were in the opposite direction, i.e. a potential increase in FCM concentrations in whelping females late in the breeding period. However, the variation in females during the early luteal period may warrant further investigation in this species.

Finally, among females, there was no relationship between any of the faecal hormone metabolites measured and litter size in our study. Litter size was generally comparable with prior reports: the average litter size at birth was 2.5 offspring, with an average of 1.7 surviving to weaning age. In prior work in managed black-footed ferrets, average litter size at birth has been documented as 3.0–3.6 offspring, with 1.2–2.4 surviving to weaning age (Williams *et al.*, 1991; Young *et al.*, 2001). We collected postpartum hormone metabolite data to test potential associations with post-whelping reproductive differences to understand if there were hormonal changes that accompanied litter size differences or if offspring did not survive. We did not detect such an association with litter size in this study, and very few females had no offspring survive the postpartum period (only one whelping female per year had no offspring survive, and these were different individuals each year; Supplementary Table S1). However, we still include this postpartum hormone metabolite data to show the lack of effect and for documentation of changes in faecal hormone metabolites by reproductive state, which may be of interest for future comparative work.

In males, we explored the role of testosterone in successfully siring litters. There were no differences in FTM concentrations between successful sires, older males who were not bred and males who bred but did not successfully sire a litter. However, males that failed to mate (either being unsuccessful in mounting females or negative sperm check) had lower FTM concentrations than all other groups. This indicates that, for the males who successfully mated but did not sire a litter, the issue is not with testosterone levels, mating behaviour, or production of sperm, per se, but potentially with sperm morphology and/or function. While female physiology may be involved in unsuccessful pregnancies, there was a particular male in this study (2022–23) that was mated with different females with positive sperm checks, but he did not sire litters in either year (Supplementary Table S1). Among the other males that were represented in both years of the study, all males that successfully sired in 2022 successfully sired again in 2023. This, along with prior work on abnormal sperm morphology in this species (Wolf *et al.*, 2000b; Howard and Wildt, 2009; Santymire *et al.*, 2019), is suggestive that malformed sperm may be associated with some of the DNW cases. While we did not detect significant differences in sperm structural defects between successful and unsuccessful sires, our sperm morphology sample size was small and analyses underpowered. Hence, it is possible failed sire cases could be driven by sperm abnormalities (e.g. apical ridge defects, Santymire *et al.*, 2019).

Finally, we found a negative association between faecal hormone metabolite concentrations and progressive days in the breeding season in males. The earlier days (Day 0 being the first female oestrus of the season) correspond to the peak mating opportunities between paired male and female black-footed ferrets. However, once females are all mated, they are no longer paired and mating opportunities come to a halt. This timeline corresponds with the declining FTM concentrations throughout the study, and is in agreement with prior work in this species (Brown, 1997; Santymire *et al.*, 2020). Further, we found that FCM concentrations similarly declined across study days, and FCM and FTM concentrations were correlated. The elevated FCM levels early in the breeding period may be preparative for a period of high expenditure as breeding activity begins (in accordance with the preparative hypothesis; Romero, 2002) and this change may be seasonal in captive animals and not necessarily associated with direct triggers or ‘stressors’ in the environment (Edwards *et al.*, 2016). Testosterone and cortisol have potential costs when secreted at elevated levels for a prolonged period of time, including negative effects on the immune system in some instances (Sapolsky *et al.*, 2000; Roberts *et al.*, 2004). Hence, it may be adaptive for males to maximize testosterone and cortisol production early in the breeding season to support reproductive physiology and engage in mating opportunities, and then reduce production later in the breeding seasons once opportunities have passed.

These findings have implications for the managed care of black-footed ferrets in the conservation breeding programme. Young *et al.* (2001) proposed that elevated cortisol may be a cause of reproductive failure in black-footed ferrets. In particular, they raised concerns about how handling during the care of these animals may elevate cortisol concentrations. While restraint did acutely increase FCMs in some individuals in that study (Young *et al.*, 2001), we find that, in our longitudinal study across the breeding season, reproductive failure is not associated with elevated FCMs in this species. We suggest that the relationship between handling and acute stress is not a general concern for managed populations of black-footed ferrets. Further, our results highlight individuals for potential targeted treatment in the breeding programme. Recent work (Santymire *et al.*, 2020) has demonstrated that vitamin E supplementation can be used to increase testosterone levels in male black-footed ferrets. We have found that males that fail to mate (those who failed to approach a female or had negative sperm checks after copulation) have lower FTMs than other groups. These males could be treated with vitamin E supplementation specifically, rather than broadly treating all males, which is more costly, time-consuming and could potentially have adverse health effects if testosterone is further elevated in high-T individuals.

A caveat of our study, and many studies working with rare or endangered populations, is the small sample size of individuals from which to draw conclusions, which is problematic due to the risk of Type II errors and the difficulties of replicating findings across studies (Brosi and Biber, 2009; Shaw *et al.*, 2021). This suggests that significant differences among groups may be missed due to the few individuals represented, and we particularly highlight this with respect to several marginal results found among female reproductive outcomes in our study. However, we did detect robust differences in FTMs between male groups, as well as replicated seasonal and reproductive state-specific patterns previously detected in this species. Within this study, there were additionally reproductive outcomes with very small sample sizes that could not be investigated more deeply, e.g. the single female that did not exhibit signs of oestrus. While this is aligned with early work that demonstrated that most female black-footed ferrets naturally undergo oestrus each breeding season (Williams *et al.*, 1991), it is difficult to understand the factors that may drive these rare failures. Similarly, litter loss was rare in our study, and it is difficult to analyse why this occasionally occurs.

In summary, we found that individuals with different reproductive outcomes displayed endocrine differences across the breeding season; in particular, successfully whelping females tended to have elevated FPM and FCM levels during late pregnancy and postpartum relative to DNW females, and males who failed to mate had reduced FTM levels. However, there were no faecal hormone differences that could be used to reliably predict reproductive failure in advance in DNW females and in males who successfully mated but failed to sire. While this limits the use of steroid hormone measures as predictive tools in this endangered species, it indicates that the causes of these reproductive failures are not driven by, or reflected in, steroid hormone levels.

## Supplementary Material

Web_Material_coaf002

## Data Availability

Data available on request.
