# Supplementary material for: Reproductive endocrinology of endangered black-footed ferrets: implications for conservation breeding
Source: Conserv Physiol. 2025 Feb 13;13(1):coaf002. doi: 10.1093/conphys/coaf002 (PMC11825693; doi:10.1093/conphys/coaf002)
Supplement: Web_Material_coaf002 [file web_material_coaf002.zip › BFF_SuppMaterials_Dec2024.pdf]

Table S1. Individual reproductive outcomes of black-footed ferrets and animal sex, age, and outcome the previous year of the study (when applicable). Reproductive outcomes for females were as follows: did not whelp (DNW), whelped (instances where no offspring survived past weaning are indicated), or no oestrus occurred. Reproductive outcomes for males were as follows: not mated (due to old age), sired, female DNW (a positive sperm check was found in the vaginal lavage after mating, but the female never gave birth to a litter), and failed to mate (either no sperm found in the vaginal lavage after mating or the male failed to approach and mount females). The number of fecal samples collected per individual per year is indicated in the last column.

Year	Animal ID	Sex	Age (years)	Reproductive outcome	Prior reproductive outcome (2022)	N fecal samples
2022	Guess	F	3	DNW		20
2022	Oma	F	3	Whelped		19
2022	Nana	F	2	Whelped		18
2022	Abuela	F	2	Whelped		23
2022	Philo	F	2	Whelped; no offspring survived		21
2022	Rachel	F	1	Whelped		21
2022	Fireworks	F	1	Whelped		22
2022	Harper	F	1	DNW		20
2022	George	M	5	Not mated		11
2022	Chestnut	M	5	Not mated		10
2022	Buzz	M	4	Female DNW		15
2022	Coconut	M	3	Sired		14
2022	Cosmo	M	2	Female DNW		17
2022	Nonno	M	2	Sired		12
2022	Trapani	M	1	Failed to mate; negative sperm check		8
2022	Howick	M	1	Sired		13
2022	Levis	M	1	Sired		15
2023	Nana	F	3	Whelped	Whelped	24
2023	Abuela	F	3	Whelped	Whelped	21
2023	Rachel	F	2	DNW	Whelped	22
2023	Fireworks	F	2	Whelped; no offspring survived	Whelped	27
2023	Harper	F	2	No oestrus	DNW	25
2023	Seagull	F	1	DNW		23
2023	Seacucumber	F	1	Whelped		19

2023	Gerri	F	1	DNW		20
2023	Coconut	M	4	Sired	Sired	17
2023	Cosmo	M	4	Female DNW	Female DNW	15
2023	Nonno	M	3	Sired	Sired	14
2023	Howick	M	2	Sired	Sired	19
2023	Levis	M	2	Sired	Sired	17
2023	LittleG	M	1	Failed to mate; behavioral		15
2023	Weatherman	M	1	Failed to mate; behavioral		15

Table S2. Results of mixed effect models analyzing the effects of reproductive outcome, reproductive state, and the outcome and state interaction effect, and Tukey post-hoc tests on reproductive outcomes by reproductive state. All models include individual ID nested within year as random effects. The main effect of reproductive outcome is relative to did not whelp (DNW) as the intercept, and reproductive states are relative to the follicular phase as the intercept. Marginally significant and statistically significant values are bolded, with # indicating < 0.10, * < 0.05, ** < 0.01, and ***<0.001.

Response variable	Parameter	Estimate ± SE	DF	t value	P value
Fecal progesterone metabolites (log ng/g)	Intercept	2.17 ± 0.10	289	21.12	0.000
	Outcome - whelp	0.14 ± 0.08	12	1.64	0.13
	Phase - oestrus	0.19 ± 0.12	289	1.56	0.12
	**Phase - early luteal**	**0.33 ± 0.12**	289	**2.84**	**0.004*** **
	**Phase - late luteal**	**0.57 ± 0.08**	289	**7.07**	**0.000*** **
	Phase - postpartum	0.11 ± 0.10	289	1.06	0.29
	Post-hoc comparisons of outcome by reproductive state				
	Follicular: whelp vs. DNW	-0.14 ± 0.08	12	-1.64	0.13
	Oestrus: whelp vs. DNW	0.03 ± 0.13	12	0.20	0.84
	Early luteal: whelp vs. DNW	-0.06 ± 0.11	12	-0.55	0.59
	**Late luteal: whelp vs. DNW**	**-0.13 ± 0.06**	12	**-2.29**	**0.04***
	**Postpartum: whelp vs. DNW**	**-0.18 ± 0.09**	12	**-2.09**	**0.06#**
Fecal oestradiol metabolites (log ng/g)	Intercept	1.64 ± 0.09	270	18.17	0.000
	Outcome - whelp	0.18 ± 0.11	12	1.57	0.14
	**Phase - oestrus**	**0.26 ± 0.14**	**270**	**1.88**	**0.06#**
	Phase - early luteal	0.19 ± 0.10	270	1.43	0.15
	**Phase - late luteal**	**0.19 ± 0.10**	**270**	**1.97**	**0.05#**
	Phase - postpartum	0.01 ± 0.12	270	0.11	0.91
	Post-hoc comparisons of outcome by reproductive state				
	Follicular: whelp vs. DNW	-0.18 ± 0.11	12	-1.58	0.14
	Oestrus: whelp vs. DNW	-0.10 ± 0.15	12	-0.67	0.51
	Early luteal: whelp vs. DNW	0.07 ± 0.14	12	0.57	0.58
	Late luteal: whelp vs. DNW	-0.03 ± 0.08	12	-0.37	0.72
	Postpartum: whelp vs. DNW	-0.03 ± 0.12	12	-0.23	0.82
Fecal cortisol metabolites (log ng/g)	Intercept	1.66 ± 0.07	282	23.20	0.000
	Outcome - whelp	0.12 ± 0.09	12	1.35	0.20
	**Phase - oestrus**	**0.20 ± 0.12**	**282**	**1.70**	**0.09#**
	Phase - early luteal	0.10 ± 0.11	282	0.88	0.38
	**Phase - late luteal**	**-0.16 ± 0.08**	**282**	**-1.94**	**0.05#**
	Phase - postpartum	-0.01 ± 0.10	282	-0.08	0.93
	Post-hoc comparisons of outcome by reproductive state				
	Follicular: whelp vs. DNW	-0.11 ± 0.09	12	-1.35	0.20
	Oestrus: whelp vs. DNW	-0.05 ± 0.13	12	-0.38	0.71

	Early luteal: whelp vs. DNW	0.15 ± 0.11	12	1.37	0.20
	**Late luteal: whelp vs. DNW**	**-0.12 ± 0.06**	**12**	**-1.95**	**0.07#**
	**Postpartum: whelp vs. DNW**	**-0.17 ± 0.09**	**12**	**-1.84**	**0.09#**
P:E ratio (log)	Intercept	0.51 ± 0.13	267	3.96	0.000
	Outcome - whelp	-0.04 ± 0.09	12	-0.49	0.63
	Phase - oestrus	-0.12 ± 0.13	267	-0.97	0.33
	Phase - early luteal	0.14 ± 0.12	267	1.18	0.24
	**Phase - late luteal**	**0.41 ± 0.09**	**267**	**4.66**	**0.000*****
	Phase - postpartum	0.15 ± 0.11	267	1.36	0.17
	Post-hoc comparisons of outcome by reproductive state				
	Follicular: whelp vs. DNW	0.05 ± 0.09	12	0.49	0.63
	Oestrus: whelp vs. DNW	0.06 ± 0.14	12	0.48	0.64
	Early luteal: whelp vs. DNW	-0.16 ± 0.11	12	-1.45	0.17
	**Late luteal: whelp vs. DNW**	**-0.12 ± 0.06**	**12**	**-1.87**	**0.09#**
	Postpartum: whelp vs. DNW	-0.14 ± 0.09	12	-1.50	0.16

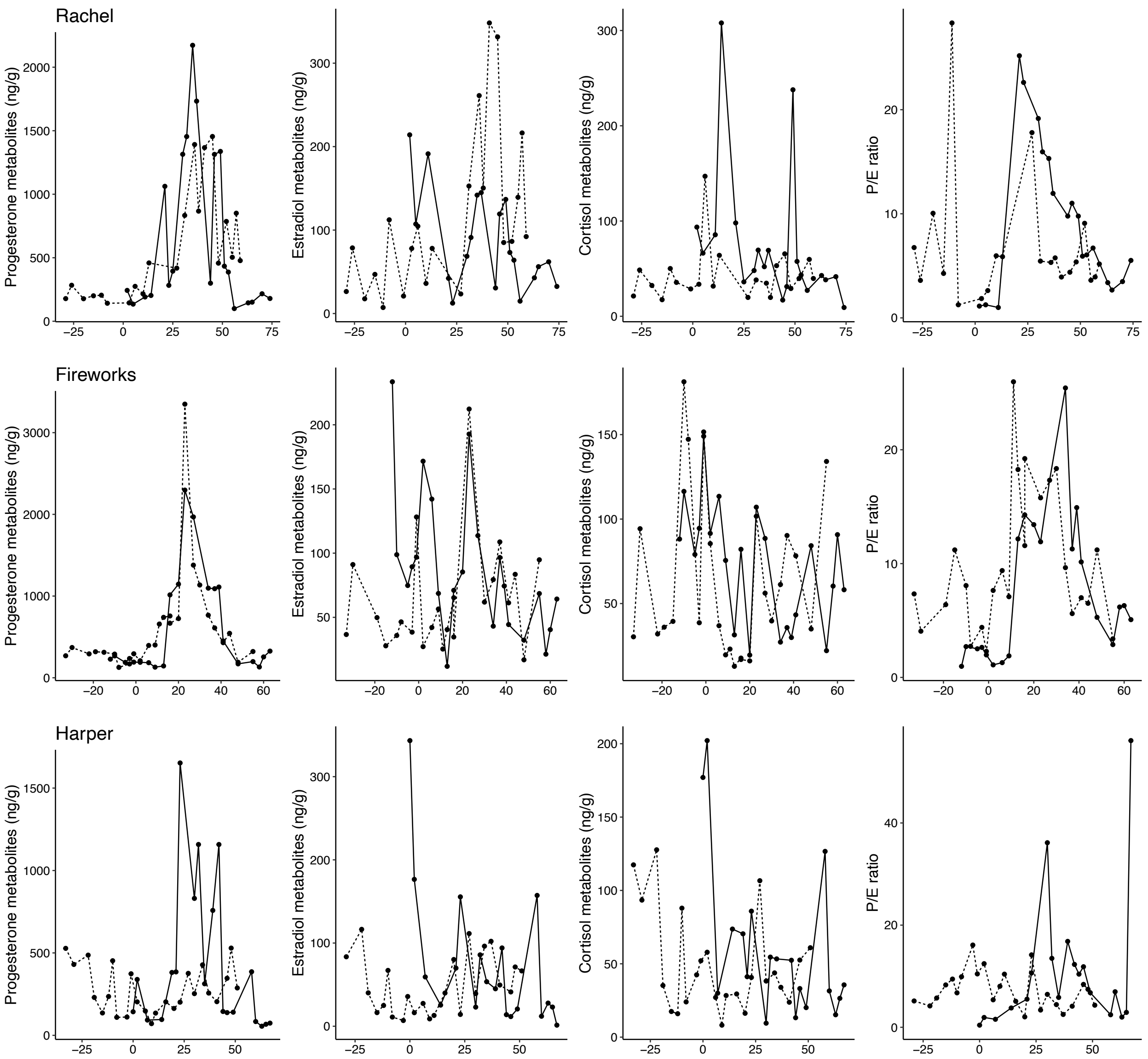

Figure S1. Fecal hormone metabolite profiles of females with different reproductive outcomes between years of the study. Solid lines are profiles from 2022, and dashed lines from 2023. In the top panel, Rachel successfully whelped in 2022 and did not whelp in 2023. In the middle panel, Fireworks successfully whelped in 2022 and 2023, but no offspring survived to weaning in 2023. In the bottom panel, Harper did not whelp in 2022 and did not enter estrus in 2023.
